# Systemic Sclerosis in a Seven-Year-Old Girl Responding to Mycophenolate Mofetil: A Case Report

**DOI:** 10.7759/cureus.82628

**Published:** 2025-04-20

**Authors:** Mohamed Alwadai, Hasan Almusaad, Rawan Yusuf, Rawah Almutawa, Shaikha Albanna

**Affiliations:** 1 Pediatrics, Salmaniya Medical Complex, Manama, BHR

**Keywords:** chronic joint pain, diffuse systemic sclerosis, limited systemic sclerosis, pediatric rheumatology, progressive dysphagia

## Abstract

Juvenile systemic sclerosis (JSSc) is a chronic, multisystem, connective tissue disease typically characterized by symmetrical fibrous thickening and hardening of the skin combined with fibrous changes in internal organs, such as the esophagus, intestinal tract, heart, lungs, and kidneys. We present the case of a seven-year-old female with a history of chest pain, irregular episodes of vomiting, and shortness of breath. Physical examination revealed hardening of the skin and darkish discoloration prominent on arms. Blood serology showed a positive anti-Scl-70 antibodies test, suggestive of JSSc. Treatment with prednisolone was initiated, followed by mycophenolate mofetil (MMF). This case highlights the importance of considering MMF in the treatment of patients with JSSc. Early diagnosis and intervention are essential in optimizing the quality of life for patients with JSSc.

## Introduction

Systemic sclerosis (SSc), also known as scleroderma, is a group of diseases that can affect multiple organs and occur at any stage of life. In children, it is referred to as juvenile systemic sclerosis (JSSc) [1]. JSSc is classified based on clinical findings, with skin sclerosis or induration serving as the major criterion, accompanied by a characteristic pattern of internal organ involvement. The condition is further categorized into subsets, including diffuse cutaneous systemic sclerosis (dcSSc), limited cutaneous systemic sclerosis (lcSSc), systemic sclerosis sine scleroderma, and overlap syndromes [2]. Compared to adult-onset systemic sclerosis, there are significantly fewer reported cases on JSSc. While systemic sclerosis affects approximately 50-300 individuals per million in the general population, JSSc accounts for only 1-10% of these cases. Epidemiological data estimate the incidence of JSSc to be between 0.27 and 2.9 cases per million children annually and its prevalence to range from one to five per million in the pediatric population [3,4].

The development of JSSc is not fully understood, but environmental and genetic factors are believed to contribute to its pathogenesis. The disease presents as a spectrum, ranging from the slowly progressing limited cutaneous systemic sclerosis to the more aggressive diffuse cutaneous systemic sclerosis [5]. Treating JSSc remains a challenge for rheumatologists due to the lack of standardized treatment protocols. Current management includes immunosuppressive therapies such as corticosteroids, methotrexate, cyclophosphamide, and mycophenolate mofetil (MMF) [6].

MMF has shown promise in managing SSc due to its relatively favorable side-effect profile. It functions by inhibiting the purine synthesis pathway, leading to reduced cell proliferation [7]. In scleroderma-related interstitial lung disease, studies suggest that MMF offers superior tolerability and a better toxicity profile compared to cyclophosphamide [8]. However, while most clinical evidence and trials evaluating MMF's efficacy in scleroderma focus on adult populations, data on pediatric cases remain limited.

Given the scarcity of pediatric cases, evidence supporting effective management strategies remains limited. This case report aims to add to the limited body of literature on JSSc, and to supplement the emerging pediatric data and highlight MMF’s potential utility in managing JSSc.

## Case presentation

A seven-year-old girl presented to the emergency department with a six-month history of multiple symptoms, including irregular morning vomiting (yellowish in color), chest pain associated with feeding (described as "a volcano is present in my upper abdomen"), and progressive skin tightening with dark discoloration, particularly on the arms. Additional symptoms included exertional shortness of breath within 10 minutes of physical activity, intermittent constipation, and weight loss of 2.5 kg over the past 45 days. The patient also experienced episodic reddish discoloration of stools.

Her past medical history was significant for coarctation of the aorta, surgically repaired via left thoracotomy and resection at one year of age. There was no known family history of hereditary diseases. She reported intermittent dizziness unrelated to positional changes, early satiety, easy fatigability, and exertional breathlessness. Additionally, she experienced generalized joint pain without muscle weakness. Her skin was painful but non-pruritic, with non-specific tenderness in the limbs.

**Figure 1:**
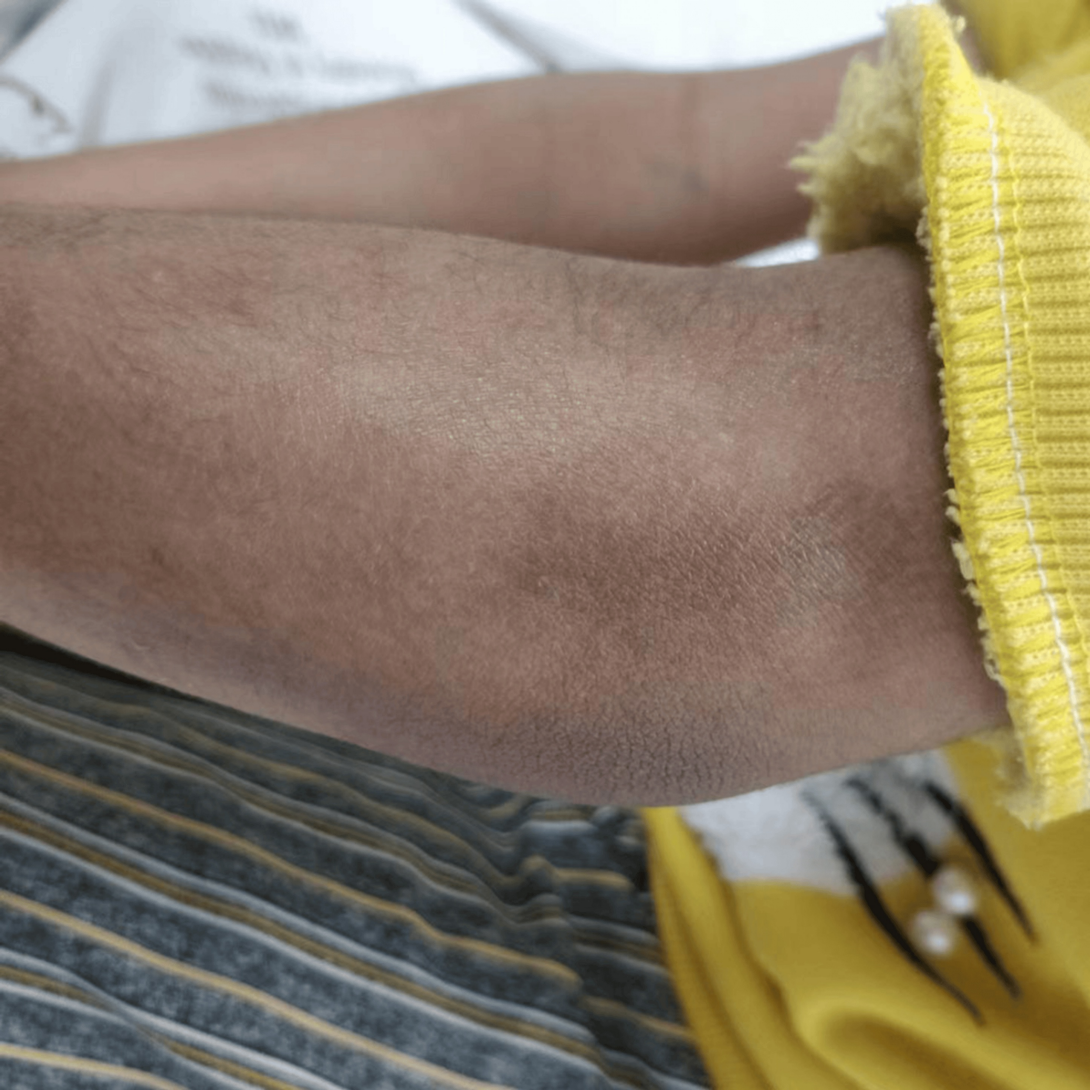
Physical examination of the patient showing a dry, hard and thick skin.

**Figure 2:**
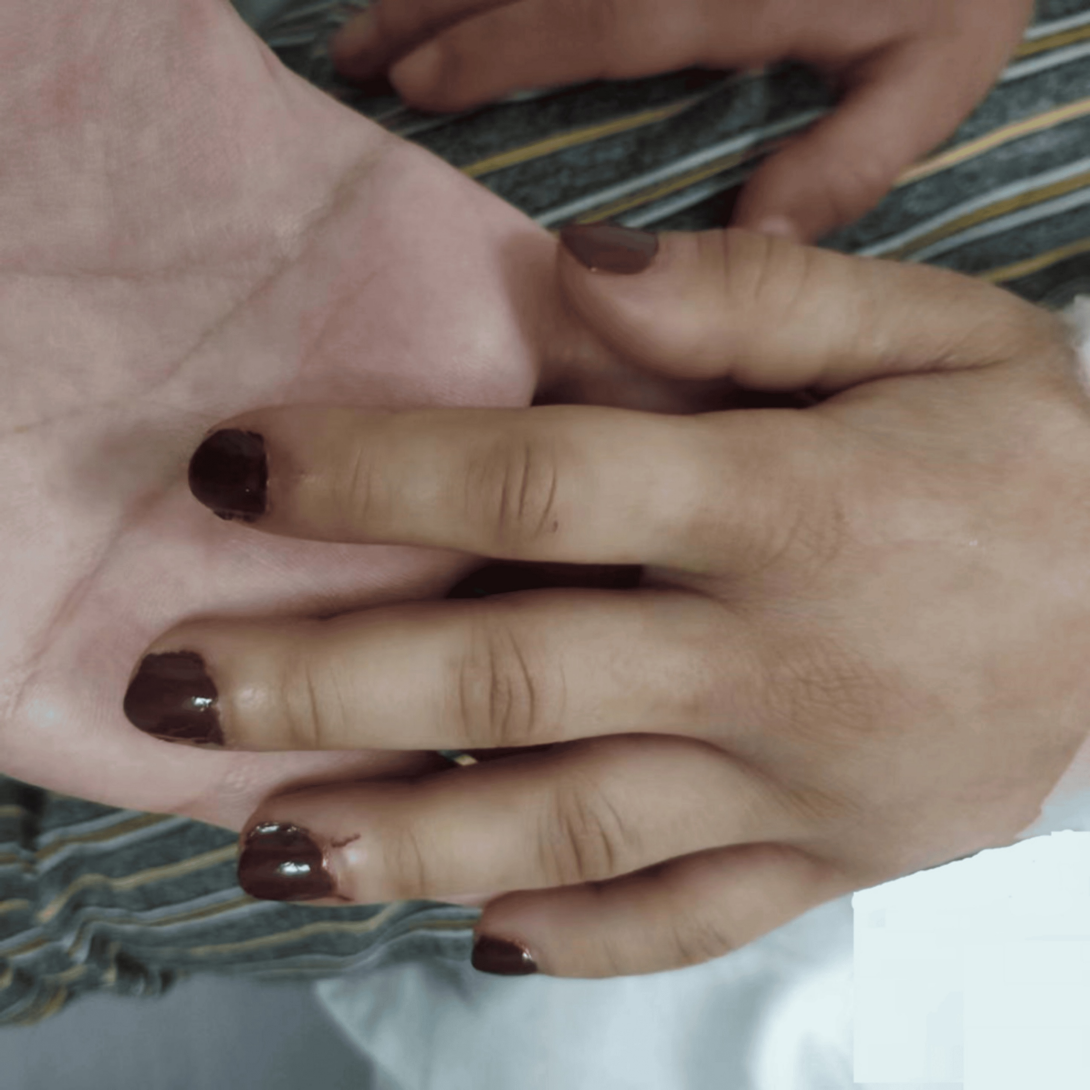
Physical examination of the patient showing thickening and swelling of the fingers.

Vital signs were within normal limits. On examination, the patient appeared thin but well-developed. Skin assessment revealed hyperpigmentation and induration, particularly on the upper extremities, chest, and back (Figure 1). The skin over the fingers and forearms was hard and tight, with restricted mobility (Figure 2). Musculoskeletal examination revealed diffuse joint tenderness without synovitis or muscle weakness. Pulmonary auscultation demonstrated mild bibasilar crackles. Cardiovascular examination revealed a normal heart rate and rhythm with no murmurs. Abdominal examination was unremarkable except for mild hepatomegaly.

**Figure 3:**
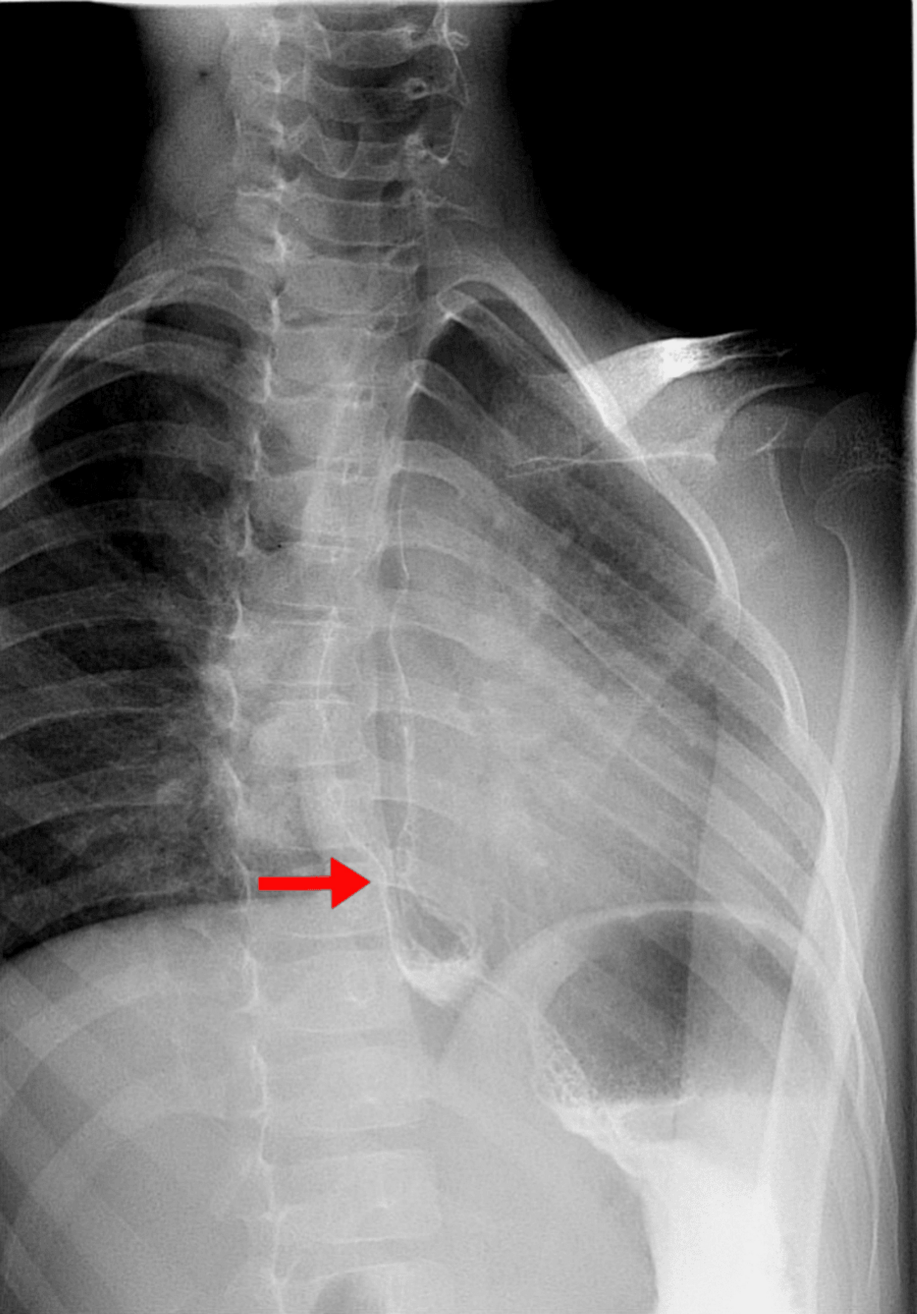
Barium swallow X-ray showing focal persistent narrowing seen within the distal aspect of the esophagus (red arrow) suggestive of fibrosis.

**Figure 4:**
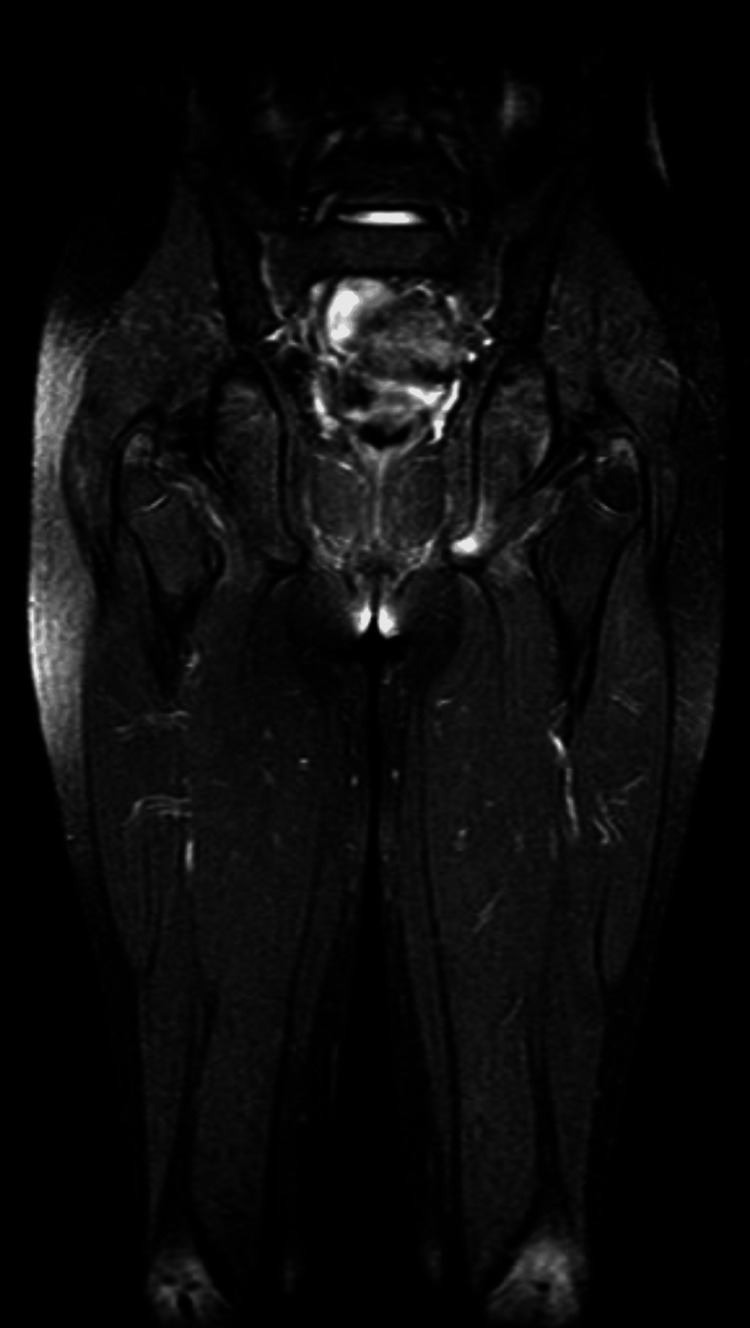
Coronal T2-weighted fat-suppressed MRI of the pelvis and proximal thighs showing diffusely hyperintense muscles, indicative of inflammatory myopathy.

Laboratory investigations revealed a positive anti-extractable nuclear antigen (SCL-70) result (normal reference: negative) (Table 1). Pulmonary function tests demonstrated a restrictive pattern on spirometry, with forced vital capacity (FVC) of 71% (normal: 80-120%), forced expiratory volume in 1 second (FEV1) of 76% (normal: 80-120%), and an FEV1/FVC ratio of 110% (normal: 75-85%). A barium meal study demonstrated focal persistent narrowing in the distal esophagus, suggestive of fibrosis (Figure 3). Hip MRI revealed diffusely hyperintense muscles indicating inflammatory myopathy (Figure 4).

**Table 1:** Laboratory tests. ESR: erythrocyte sedimentation rate.

Test	Result	Normal range
Hemoglobin	9.0 g/dL	11.9–14.8 g/d
White blood cells (WBC)	6.75 × 10³/µL	4.1–10.4 × 10³/µL
Platelets	210,000/µL	176.9–381.3 × 10³/µL
ESR	60 mm/hr	0–10 mm/hr
Urea	13 mg/dL	10–40 mg/dL
Creatinine	0.5 mg/dL	0.45–0.81 mg/dL
Creatine kinase	697 U/L	21-215 U/L
Lactate dehydrogenase	356 U/L	100-300 U/L
Antinuclear antibody	Positive	Negative
Anti-Scl-70 antibody	Positive	Negative
Anticentromere antibody	Negative	Negative
C3 complement level	118 mg/dL	90-180 mg/dL
C4 complement level	32 mg/dL	10-40 mg/dL

The constellation of clinical findings, serologic testing, and imaging confirmed the diagnosis of juvenile systemic sclerosis (JSSc). Given the multisystem involvement, a multidisciplinary approach was recommended, involving cardiology, pulmonology, dermatology, gastroenterology, and physical medicine for comprehensive management.

Following a discussion with the patient's parents, treatment was initiated with prednisolone 5 mg daily and mycophenolate mofetil (MMF) 350 mg twice daily. The MMF dose was subsequently increased to 500 mg twice daily, while prednisolone was tapered gradually. Supportive management included magnesium hydroxide, folic acid, vitamin D, omeprazole, and physiotherapy.

At her two-week follow-up, she demonstrated significant clinical improvement, including increased energy levels, reduced heartburn, improved appetite, and decreased swallowing difficulties. Skin pigmentation lightened, and areas of sclerosis, particularly on the upper arms and chest, showed softening. Subsequent follow-ups confirmed sustained symptom control, with the patient reporting improved daily functioning and overall well-being.

## Discussion

Systemic sclerosis (SSc; scleroderma) is a chronic multisystem disease characterized by skin thickening and internal organ involvement [1]. In children, it is referred to as juvenile systemic sclerosis (JSSc), which is classified into diffuse cutaneous systemic sclerosis (dcSSc), limited cutaneous systemic sclerosis (lcSSc), overlap syndromes, and systemic sclerosis sine scleroderma [5]. Our patient fulfilled one major criterion and seven minor criteria, classifying her under dcSSc.

Children with JSSc rarely present with early visceral organ involvement; however, our patient exhibited gastrointestinal, respiratory, and musculoskeletal symptoms, likely due to a delay in seeking medical care. Gastrointestinal involvement is reported in 30% to 74% of JSSc cases [9] and was the most significant complaint in our patient. A high titer of anti-topoisomerase I (Scl-70) antibodies further supported the diagnosis of JSSc. Treatment of JSSc requires a multidisciplinary approach, including patient and caregiver education, psychological and social support, skincare, avoidance of cold exposure, exercise, pharmacologic therapy, and surgical intervention if necessary [10]. 

Immunosuppressive therapy remains the mainstay of treatment, with corticosteroids, methotrexate, cyclophosphamide, and mycophenolate mofetil (MMF) being commonly used agents [11]. MMF has emerged as a promising treatment option due to its favorable efficacy and safety profile, particularly in scleroderma-related interstitial lung disease and severe skin involvement [12]. Studies have shown MMF to be well tolerated compared to cyclophosphamide, with fewer toxic effects and better long-term outcomes in scleroderma-related lung disease and skin involvement [13].

While methotrexate is often considered a first-line immunosuppressive agent in the treatment of juvenile systemic sclerosis (JSSc), especially in milder or skin-limited disease, treatment decisions in JSSc are highly individualized and often guided by the severity of organ involvement, clinician experience, and emerging evidence from adult studies due to the absence of pediatric-specific treatment guidelines [5,6,10,12].

Our patient presented with early visceral involvement, including restrictive lung disease, esophageal fibrosis, and inflammatory myopathy, prompting a multidisciplinary team to initiate low-dose prednisolone for initial control and to minimize potential steroid-related toxicity in a pediatric patient. The dose was escalated alongside mycophenolate mofetil (MMF), which was selected based on its favorable safety profile, growing evidence of efficacy in scleroderma-related interstitial lung disease, and tolerability in children [6-8,13].

Currently, there is no universally accepted treatment protocol for JSSc [5,6,10,12,13]. Consensus-based recommendations suggest tailoring therapy based on organ involvement and tolerability. A recent evidence-based review also acknowledges that both methotrexate and MMF may be used depending on clinical context [6].

## Conclusions

This case highlights the significant role of MMF in the management of JSSc, showing its efficacy and tolerability in controlling disease progression. Furthermore, the case highlights the importance of an early diagnostic approach that integrates clinical assessment and advanced laboratory testing to ensure timely diagnosis and appropriate management. Given the multisystem involvement of JSSc, a multidisciplinary approach is essential to address systemic manifestations, optimize treatment outcomes, and improve overall quality of life. Continued research and additional case studies are essential to expand the understanding of these rare cases.
